# Enhancing financial security of female sex workers through a community-led intervention in India: Evidence from a longitudinal survey

**DOI:** 10.1371/journal.pone.0223961

**Published:** 2019-10-22

**Authors:** Sangram Kishor Patel, Saradiya Mukherjee, Bidhubhusan Mahapatra, Madhusudana Battala, Matangi Jayaram, Sameer Kumta, Yamini Atmavilas, Niranjan Saggurti

**Affiliations:** 1 HIV and AIDS Program, Population Council, New Delhi, India; 2 Bill & Melinda Gates Foundation, New Delhi, India; USC Keck School of Medicine, Institute for Global Health, UNITED STATES

## Abstract

**Introduction:**

Community-led organizations (COs) have been an integral part of HIV prevention programs to address the socio-economic and structural vulnerabilities faced by female sex workers (FSWs). The current study examines whether strengthening of community-led organizations and community collectivization have been instrumental in reducing the financial vulnerability and empowering FSWs in terms of their self-efficacy, confidence, and individual agency in India.

**Data and methods:**

This study used a panel data of 2085 FSWs selected from 38 COs across five states of India. Two rounds of data (Round 1 in 2015 and Round 2 in 2017) were collected among FSWs. Data were collected both at CO and individual level. CO level data was used to assess the CO strength. Individual level data was used to measure financial security, community collectivization, and individual empowerment.

**Results:**

There was a significant improvement in CO strength and community collectivization from Round 1 to Round 2. High CO strength has led to improved financial security among FSWs (R2: 85% vs. R1: 51%, AOR: 2.5; 95% CI: 1.5–4.1) from Round 1 to Round 2. High collective efficacy and community ownership have improved the financial security of FSWs during the inter-survey period. Further, the improvement in financial security in the inter-survey period led to increased or sustained individual empowerment (in terms of self-confidence, self-efficacy, and individual agency) among FSWs.

**Conclusions:**

Institutional strengthening and community mobilization programs are key to address the structural issues and the decrease of financial vulnerability among FSWs. In addition, enhanced financial security is very important to sustain or improve the individual empowerment of FSWs. Further attention is needed to sustain the existing community advocacy and engagement systems to address the vulnerabilities faced by marginalized populations and build their empowerment.

## Introduction

HIV prevention programs around the globe have used community mobilization strategies as an important mechanism to address social, political, and legal vulnerabilities among female sex workers (FSWs) by encouraging collectivization [1]. The process of community mobilization begins when sex workers see themselves as part of a community and work together as a group [2]. A strong sense of collectivization helps them to confront multiple structural barriers, resulting in both individual and collective empowerment and improved social cohesion [3–6]. Other way, community collectivization works as a mechanism to popularize and enhance sex workers’ safe sex practices, and demand for basic rights and quality services at the ground level [7, 8]. Community mobilizing interventions around the globe bear evidence of successes in increased condom use [1, 7–10], improved service access and quality [5, 6, 11] and promoting uptake of HIV counselling and testing [12]. Additionally, involvement of community groups and strengthening community systems through leadership, training, advocacy and demand creation can generate a strong community structure for linking communities to services [13]. Evidence from Uganda suggests that strengthening community systems could alleviate barriers of community-based HIV programs by creating an enabling environment for communities to engage in service delivery and uptake [14].

The Sonagachi community mobilization model under the Durbar Mahila Samanwaya Committee (DMSC) program has been shown to have a secondary impact on economic strengthening and is a highly appreciated FSW interventions in India [9, 15]. Abundant literature has cited economic constraint and poverty as the prime motives for entering sex work, which elevates the risk of acquiring HIV in multiple ways [3, 16]. Economic disadvantage limits the negotiation power of FSWs with clients—who frequently offer higher prices for sex without a condom—and low self-efficacy to bargain for safe sex with clients makes them more vulnerable toward HIV risk [17]. A few studies have tested if economic upliftment improved the HIV related risks of FSWs. An FSW intervention in an urban slum in Kenya that introduced condoms, group-based loans, and business training and mentorship resulted in fewer sex partners among participants and increased condom use with regular partners [18]. A microenterprise intervention conducted among sex workers in Chennai, India found the intervention successful in generating financial security through alternative income sources, which was also associated with reducing FSW HIV risk [19]. Additionally, the community mobilization strategy of a program reducing financial vulnerability among sex workers was explored in a study conducted in southern India, which established a positive relationship between community collectivization and improved financial conditions among sex workers [20].

**Fig 1:**
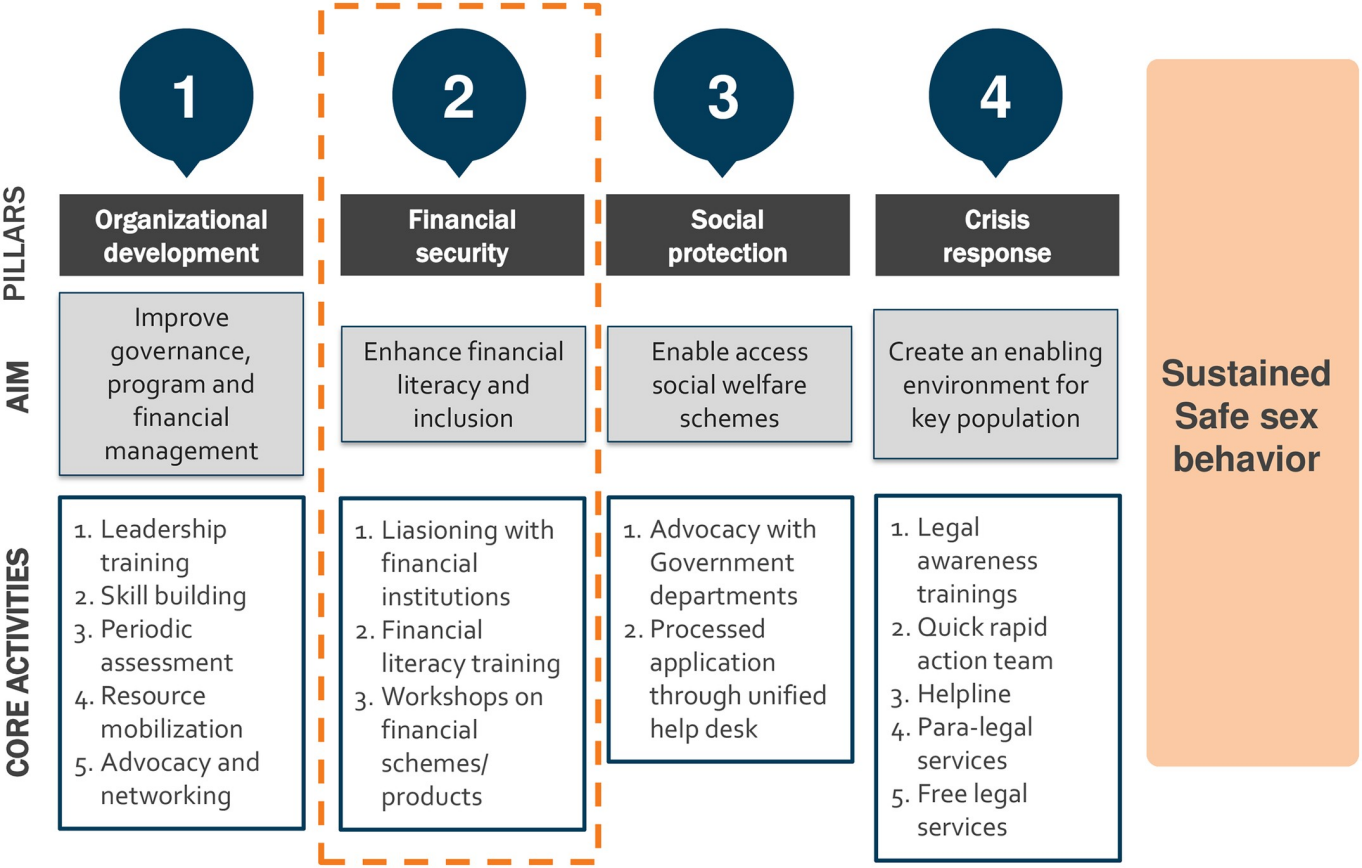
Avahan-3 community led-program model depicting financial security as an important pillar to reduce vulnerabilities among FSWs.

The first two phases of the Avahan program in India, funded by the Bill & Melinda Gates Foundation (BMGF) were very successful interventions [21, 22]. The learnings from the first two phases of the Avahan program (Phase 1: 2003–2008, Phase 2: 2009–2013) describes how they were highly successful in reducing HIV infection by improving condom use, up-take of HIV prevention services, and addressing various structural barriers [23, 24]. The Avahan Phase 3 program is the last phase of the Avahan program (2014–2017), was designed and implemented by Swasti. In the last phase of the program a special emphasis was given towards increasing the financial security of FSWs by providing financial literacy assistance—supporting them to open saving bank accounts and facilitating the process of financial investments through community organizations (COs) (Fig 1). This phase also provided special attention to building the capacity of COs, with the hypothesis that stronger COs would be able help members address the socio-economic and structural vulnerabilities they faced. In this context, it is useful to explore the dynamics between how community collectivization and institutional strengthening work towards enhancing financial security among FSWs, which in turn positively uplifts individual empowerment through self-efficacy, individual agency, and self-confidence among FSWs. Previously, this complex relationship between improved financial security and individual empowerment has not been explored adequately. Therefore, the current study aims to fulfill these gaps by examining how FSWs’ community collectivization and the institutional strengthening of COs influence the financial security and individual empowerment of FSWs. The specific objectives of the study are to a) assess the financial security of FSWs by community collectivization indicators and CO strength in India; b) establish the relationship between financial security and individual empowerment (e.g. self-efficacy, individual agency, and self-confidence) of FSWs in India.

## Data and methods

### Data

The study used data collected from a panel of 2085 FSWs across five states of India (Karnataka, Maharashtra, Tamil Nadu, Andhra Pradesh, and Telangana) from two survey rounds conducted in August 2015 (Round-1) and December 2018 (Round-2). For sampling purposes, Andhra Pradesh and Telangana were considered one entity, as they were one state when the study was being planned. Within each state, a three-stage sampling process was used to select the respondents. A total of 38 COs were selected in the first stage, three clusters (geographical area of about 250–300 FSWs) were selected within each CO in the second stage, and 30 FSWs were randomly selected from each selected cluster in the third stage. Prior to the start of the Round-1 survey, a minimum sample size of 873 (rounded off to 900) per state was estimated to detect a change of 10 percentage points from a Round-1 value of 50% with a 5% level of significance, 90% power, design effect of 1.7, and loss to follow-up rate of 40%. About 42% of FSWs surveyed at Round-1 could not be contacted during the Round-2 survey. Data for FSWs from one CO area covered during Round-1 could not be interviewed at the time of the Round-2 because their CO leadership did not agree to participate in the survey. The other reason for loss to follow-up was the movement of FSWs for sex work practice outside the survey area at the time of survey. A comparison of the Round-1 profile of FSWs who were interviewed and who could not be interviewed at Round-2 suggested that their profile was almost similar (in Table 1). Data were collected at both CO and individual levels; CO level data was used to assess performance of COs, whereas individual data used to measure vulnerabilities and HIV risk behaviors. All interviews were conducted by trained female investigators with verbal and written skills in the local language of each state. A structured survey schedule was used for collecting data using face-to-face interview techniques.

**Table 1:** Socio-demographic profile of female sex workers and CO level indicators in India: Baseline (Round-1, 2015) vs endline (Round-2, 2017).

	Round-I	Round-II
	% (n) or Median	% (n) or Median
***Socio-demographic characteristics of FSWs***		
**Median age**	35.0	37.0
**Age**		
< 30 years	20.9 (435)	12.5 (260)
30–39 years	53.7 (1119)	48.9 (1019)
≥ 40 years	25.5 (531)	38.6 (806)
**Education**		
No formal education	42.0 (875)	41.3 (860)
Having formal education	58.0 (1210)	58.7 (1225)
**Marital status**		
Currently married	64.0 (1334)	63.6 (1325)
Never married	7.1 (148)	3.9 (82)
Widowed/deserted/separated/ divorced	28.9 (603)	32.5 (678)
**Typology for sex work**^#^		
Home-based	51.3 (1069)	43.0 (895)
Brothel and lodge-based	27.0 (563)	26.6 (556)
Street/public places	21.7 (453)	30.4 (634)
**Living arrangements**		
Living alone	21.5 (448)	10.7 (223)
With husbands/parents	62.0 (1292)	78.1 (1629)
With others/sex workers	16.5 (345)	11.2 (233)
**Total**	**100.0 (2085)**	**100.0 (2085)**
***CO Level indicators***		
**Median duration of FSW CO formation**	9.0	11.0
**CO strength**		
Low	50.0 (19)	13.2 (05)
High	50.0 (19)	86.8 (33)
**Total**	**100.0 (38)**	**100.0 (38)**

Note:

^#^Typology for sex work includes (i) Home-based sex work: Women who provide paid sexual services at homes (sex-worker’s own home or client’s home) or rented rooms chosen by clients; (ii) Brothel or lodge-based sex work: Women who are paid for sex, residing and/or soliciting clients from a fixed place (brothels or lodges) or any similar places like hotel/dhaba/bar/night club/massage parlour; and (iii) Street/public places-based sex work: Women who are paid for sex and cruise from one place to another, soliciting clients at any suitable public place (pick-up points, highway, road, bus stand, garden, vehicle etc.).

### Ethics statement

Institutional review boards of the Population Council and Sahara, Center for Residential Care and Rehabilitation reviewed and approved the study procedure and tools. Prior to starting interviews, respondents were explained the study procedures, risks, and benefits associated with their participation and written consent was obtained from respondents who could read and write. The consent process was explained in the presence of a witness (either program staff or fellow sex worker) and verbal consent was taken for participants who could not read and write. All the interviews were held in a private location specifically reserved for the survey or in a location convenient to the study participants.

### Measures

The study assessed socio-demographic and sex work-related characteristics (age, education, marital status, living arrangement, and typology of sex work) using single-item questions. The key independent measures used in the analysis were community mobilization indicators collected during Round-2, such as collective efficacy (low/high), collective agency (low/high), collective action (low/high), and community ownership (low/high). Similarly, the key outcome measures for this study are individual agency, self-efficacy, and self-confidence among FSWs. Measures of individual agency, self-confidence, and self-efficacy were created based on a series of statements which were read to respondents and their opinions were sought using a four-point Likert scale. These statements used to assess collective efficacy, collective agency, collective action, community ownership, individual agency, self-efficacy, and self-confidence were taken from previous research implemented in India among FSWs [7, 25].

This study measured financial security in two ways. First, financial security was measured as a composite index and used as an outcome variable. Secondly, financial security was measured through degree of change from Round-1 to Round-2 and both were used as a predictor and outcome variable in the analysis. An aggregated score of financial security was created by adding single item questions on whether FSWs had a savings account in the bank/post office, had invested in saving schemes, made investments in insurance products (life, health, accidental etc.), had an alternative source of income besides sex work, and had not taken any loan from informal sources. A median split was used to divide the aggregated score into two categories. FSWs who had a score above the median level were considered to have high financial security (coded as 1), otherwise they were considered to have low financial security (coded at 0). In addition, a composite measure for “degree of change in financial security” was used: remained low (remained in low financial security in both rounds); worsened (changed to low financial security in Round-2 from high financial security in round1); sustained high (remained in high financial security in both rounds); and improved (improved to high financial security in Round-2 from low financial security in Round-1).

The key organizational level predictor is a measure of CO strength. The CO strength assessed performance of COs across six domains critical for any organization’s functionality and sustainability: governance, project management, financial management, program monitoring, advocacy, networking, and resource mobilization. Overall, 32 indicators from all these domains were used to compute the CO strength score. Each dimension was assigned equal weight and indicators within each dimension were also assigned equal weight. The weighted score was divided into two groups based on the median cut-off; COs who scored below median were considered to have low CO strength, while above median was considered high CO strength. Similarly, other CO level measures were also used in the analysis: targeted interventions (TI) status (Non-TI and TI); size of CO (≤1200 and >1200 members); and duration of CO formation (<5 years and ≥5 years).

### Data analysis

Frequency and bivariate analyses were conducted to determine the distribution and examine the strength and association of socio-demographic characteristics, community mobilization, individual agency, and financial security among FSWs. The respective p-values for the bivariate analyses were calculated through a chi-square test in the panel data. To assess the relationships of the degree of financial security with community mobilization, CO and individual level indicators, adjusted odds ratios (AOR), and their 95% confidence intervals (CI) were calculated through multilevel logistic regression and multilevel ordered logistic regression adjusting for socio-demographic characteristics. The multilevel logistic regression analysis (with CO level clustering) employed here (in Tables 2 and 3) is a popular and widely known statistical method use to analyze a dataset in which there are one or more independent variables that determine an outcome, and the outcome is measured with a dichotomous variable (in which there are only two possible outcomes, e.g., success/failure or yes/no or died/lived).

**Table 2:** Financial security as reported by female sex workers in India, 2015–2017.

	Round-1	Round-2	Change from R1 to R2
Indicators	%(n)	%(n)	AORs 95% CI
Other income besides sex work^	53.2 (1109)	70.6 (1471)	2.15 (1.85–2.50) ***
Having a savings account in bank/post-office	70.1 (1462)	84.0 (1751)	2.07 (1.74–2.44) ***
Invested in saving schemes	32.7 (681)	61.0 (1271)	3.25 (2.82–3.74) ***
Invested in insurance	13.2 (275)	44.0 (917)	6.74 (5.60–8.11) ***
Loan from formal sources	4.3 (89)	5.8 (121)	1.21 (0.89–1.64)
Financial security Index	49.0 (1042)	81.7 (1704)	5.04 (4.28–5.93) ***

Note: CI: Confidence Interval; AOR: Adjusted Odds Ratio; AORs are adjusted for age, education, marital status, typology, living arrangements; AORs are derived from multilevel logistic regression models at CO and individual level;

*** indicate significance at 1%.

^Other income besides sex work: FSWs who have reported other income besides sex work have the following sources/jobs/works engaged besides sex work. Other jobs/works: Daily Labor, House maid, Petty shop/vendor, Private salaried job, Government salaried job, Massage parlor, Others—Specify (tailoring, agricultural work and factory work etc.)

**Table 3:** Association between CO level indicators with financial security among female sex workers in India, 2015–2017.

	Financial security		
	Round-1	Round-2	Change from R1 to R2
Community-led Organization level indicators	% (n)	% (n)	AOR (95% CI)
**CO strength**			
Low	48.7 (471)	53.2 (107)	
High	51.1 (571)	84.8 (1597)	2.46 (1.47–4.14)***
**TI status**			
Non-TI	41.7 (217)	73.5 (383)	
TI	52.8 (825)	84.5 (1321)	1.04 (0.72–1.50)
**Size of CO**			
≤1200	37.8 (177)	67.1 (314)	
>1200	53.5 (865)	86.0 (1390)	1.83 (1.28–2.61)***
**Duration of CO formation**			
<5 years	52.7 (347)	72.8 (479)	
≥5 years	48.7 (695)	85.8 (1225)	2.82 (2.03–3.90)***

Note: CI: Confidence Interval; AOR: Adjusted Odds Ratio; AORs are adjusted for age, education, marital status, living arrangements, typology and state. AORs are derived from multilevel logistic regression models at CO and individual level;

*** indicate significance at 1%.

**Fig 2:**
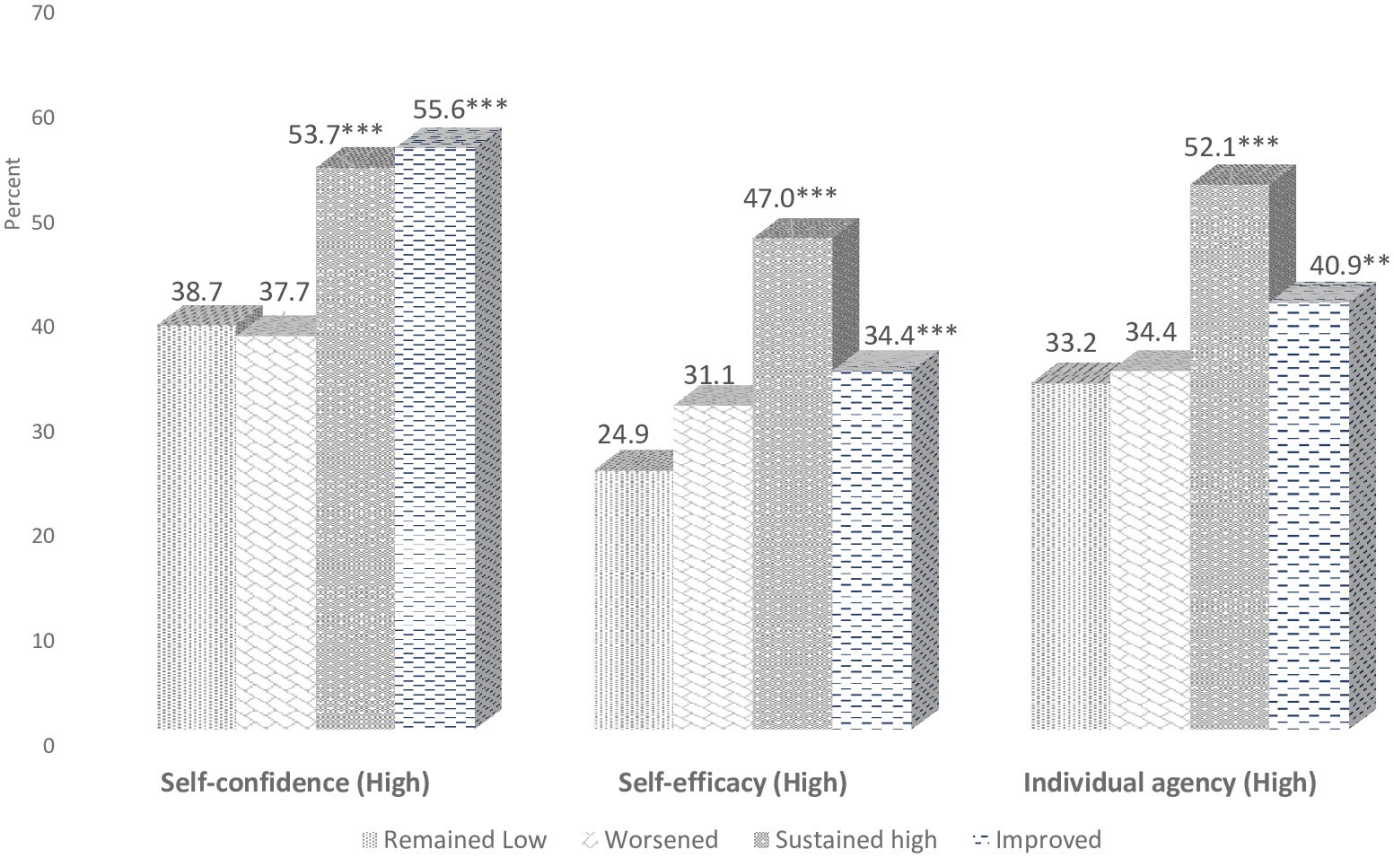
Adjusted percentages of empowerment indicators (e.g. self-confidence, individual agency and self-efficacy) by degree of change in financial security in Round-2 (2017) among female sex workers in India. Note: AOR: Adjusted Odds Ratio; AORs are adjusted for age, education, currently married, typology and living arrangements; Adjusted percentages are derived from the logistic regression models; ** and *** indicate significance at 5% and 1%.

The second regression model (with CO level clustering) used here (in Table 4) is multilevel ordered logistic regression (or proportional odds model), is an ordinal regression model-that is, a regression model for ordinal dependent variables-first considered by Peter McCullagh [26]. For example, suppose one question here is to be answered by a choice among “remained low”, “worsened”, “sustained high” and “improved”, and the purpose of the analysis is to see how well these responses can be predicted by the responses to other questions, then ordered logistic regression may be used. It can be used as an extension of the logistic regression model that applies to dichotomous dependent variables, allowing more than two (ordered) response categories. Additionally, adjusted percentages were also calculated based on the AORs (in Fig 2). All analyses were conducted using STATA software (version 13.2).

**Table 4:** Association between degree of change in financial security and community mobilization indicators in Round-2 (2017) among female sex workers in India.

	Degree of change in financial security				
	Remained Low	Worsened	Sustained high	Improved	
					AOR (95% CI)
**Collective efficacy**					
Low	15.4 (61)	13.9 (55)	40.5 (160)	30.1 (119)	
High	9.5 (161)	6.2 (104)	42.8 (723)	41.5 (702)	1.54 (1.24–1.92) ***
**Collective agency**					
Low	12.1 (124)	7.1 (73)	41.0 (421)	39.9 (410)	
High	9.3 (98)	8.1 (86)	43.7 (462)	38.9 (411)	1.03 (0.84–1.29)
**Collective action**					
Low	11.7 (71)	7.8 (47)	37.6 (228)	42.9 (260)	
High	10.2 (151)	7.6 (112)	44.3 (655)	37.9 (561)	0.93 (0.73–1.19)
**Community ownership**					
Low	12.8 (155)	10.2 (123)	40.2 (485)	36.8 (444)	
High	7.6 (67)	4.1 (36)	45.3 (398)	42.9 (377)	1.50 (1.22–1.84) ***

Note: CI: Confidence Interval; AOR: Adjusted Odds Ratio; AORs are adjusted for age, education, currently married, typology and living arrangements; AORs are derived from multilevel ordinal logistic regression models at CO and individual level;

*** indicate significance at 1%.

## Results

Of the panel sample in Round-2 (2017), the median age of the FSWs was around 37 years. At Round-2, nearly half of the FSWs included in the study were between ages 30–39 years (49%), over half of them currently married (64%), literate (59%) and living with family members/ husband (78%) (Table 1). There is very marginal variation observed in the distribution of background characteristics of FSWs in Round-2 compared to Round-1 expect the living arrangements. Median duration of CO formation was around 11 years at Round-2.

The results on financial security (Table 2) show that the likelihood of having high financial security among FSWs has increased by five times from Round-1 to Round-2 (R2: 82% vs. R1: 49%, AOR: 5.0; 95% CI: 4.3–5.9). The odds of investment in insurance has significantly improved among FSWs by more than 6 times during the inter-survey period (R2: 44% vs. R1: 13%), followed by investment in saving schemes (3 times, R2: 61% vs. R1: 33%), having a savings account in the bank/post office (2 times, R2: 84% vs. R1: 70%), and other income besides sex work (2.2 times, R2: 71% vs. R1: 53%), respectively.

The results on financial security (Table 3) also show that the likelihood of having high financial security among FSWs belonging to high strength COs increased by 2.5 times from Round-1 to Round-2 (R2: 85% vs. R1: 51%, AOR: 2.5; 95% CI: 1.5–4.1). The odds of having high financial security among FSWs belonging to COs with more than 1200 members increased by 2 times in the inter-survey period (R2: 86% vs. R1: 54%, AOR: 1.8; 95% CI: 1.3–2.6). Similarly, the likelihood of having high financial security among FSWs belonging to the COs that formed 5 or more years ago has improved by nearly 3 times from Round-1 to Round-2.

For FSWs with high collective efficacy, the odds of improved financial security versus the combined (remained low, worsened, and sustained high in the inter-survey period) financial security are 54% higher than for FSWs with low collective efficacy, given the other variables are held constant (Table 4). Similarly, for FSWs with high community ownership, the odds of improved financial security versus the combined (remained low, worsened, and sustained high) financial security are 50% higher than for FSWs with low community ownership.

The adjusted percentages were calculated for the degree of financial security with various empowerment indicators based on the adjusted odds ratios. The adjusted percentage of having high self-confidence is significantly higher among FSWs with those who have sustained (54% vs. 39%) and improved (56% vs. 39%) their high financial security status in the inter-survey period compared to those whose financial security remained low (Fig 2). The adjusted percentage of having high self-efficacy is significantly higher among FSWs with those who have sustained (47% vs. 25%) and improved (34% vs. 25%) their high financial security status in Round-2 compared to Round-1. Similarly, the adjusted percentage of having high individual agency is significantly higher among FSWs with those who have sustained (52% vs. 33%) and improved (41% vs. 33%) their high financial security status in the inter-survey period.

## Discussion

The study findings highlight that institutional strengthening of COs and community collectivization are key to enhancing the financial security of FSWs. In addition, the study is unique in explaining how the financial security of FSWs is strongly influencing the individual empowerment of FSWs (in terms of individual agency, self-efficacy, and self-confidence of FSWs). The findings of this study are in line with the findings of the earlier studies, which have shown a strong relationship between enhanced community collectivization, safer sex practices, and increased financial security among FSWs in India [7, 8, 11, 20, 27]. Additionally, evidence from other studies have well established the positive relationship between role in personal finance behavior with enhanced self-efficacy and self-confidence among marginalized population [28, 29]. The research on institutional strengthening in terms of CO strength in establishing a strong relationship with FSW’s financial security have not been well established. Our study findings are instrumental in showing these relationships. The Avahan Phase 3 program built the capacity of COs by focusing largely on a participative approach to CO leadership and second-line leaders on governance, program management, financial management, resource mobilization, advocacy, and networking. Additionally, unified help desks were set up to help FSWs in availing various services related to financial planning, financial literacy, and on financial schemes. The study findings suggest that the financial security of FSWs improved significantly with the improvement in CO strength during the inter-survey period. The enhancement of financial security associated with CO strength was also well supported by findings from the duration of CO formation and size of CO (number of FSWs covered by the CO). It is expected that as the COs grow older, their outreach services spread to a greater number of FSWs and the bonding and trust between COs and FSWs improves further, which ultimately helps reduce FSWs’ vulnerability [30].

Study findings also show that high collective efficacy and high community ownership have been instrumental in improving the degree of financial security among FSWs in the inter-survey period. The findings are comparable to another study in Southern India, which showed that strong community collectivization is one of the key components to enhance financial security among FSWs [20]. As the program reached community members, their knowledge, access, and availing the financial services and savings in formal sources increased, which in turns reduce their vulnerability. It can also be interpreted that increased community collectivization and ownership among the community members led to a smooth implementation of the program, and in turns it is expected that such programs will be sustained and improved over time [31]. Unfortunately, no significant associations were found with the degree of change in financial security (from Round-1 to Round-2) with collective agency and collective action. Further in-depth analysis on the specific components of these two broad community mobilization indicators would have very useful.

The findings of the study on the relationship between financial security and individual empowerment of FSWs add value to the current literature, which had not been researched extensively in India. This study highlights that the degree of change in financial security has positive influence on the FSWs’ self-efficacy, self-confidence, and individual agency. Previous studies have also revealed that better financial security increases the self-confidence of high-risk populations [1, 32]. Although, the relationship between self-efficacy, self-confidence and individual agency with financial behavior have also been well established among the women and adolescent’s population in earlier studies [33–35]. This study adds further to the existing literature by showing that improved and sustained financial security are also associated with the individual agency as well as self-efficacy of condom use and service utilization among FSWs. While the study findings offer several important understandings on institutional strengthening, community collectivization, and its association with FSWs’ empowerment and financial security, they must be cautiously interpreted with certain limitations. The financial security, community collectivization, and individual empowerment indicators are self-reported, which may be susceptible to social desirability and recall biases. Second, the study findings are only representative to the FSWs who are members of the selected COs of Avahan Phase 3 program areas, hence these findings may not be generalizable to FSWs in other communities in India. Third, it would have been useful to the study if the information on community mobilization and selected empowerment indicators as used here should have been collected at both round of surveys.

We can conclude from the study that institutional strengthening and community mobilization programs are prime to address the structural-level issues and the decrease of vulnerabilities (including financial vulnerability) among FSWs. In addition, enhanced financial security is very important to build the self-confidence and individual empowerment of FSWs. It adds further that COs provide a podium for FSWs to empower themselves with information and prospects that can enhance their financial security. Therefore, being part of a CO system helps FSWs become financially more secure and hence, enables them to improve their self-confidence, self-efficacy, and individual agency over time. Further attention is needed to sustain the existing community advocacy and engagement systems so that COs can continue to work on addressing the vulnerabilities faced by marginalized populations and build their empowerment. Additionally, large-scale livelihood and economic strengthening interventions are required among the key populations to reduce the economic vulnerabilities and sustain safe sex practices that reduce HIV risk.

### Funding statement

This paper was written as part of Population Council activities on the Avahan Phase 3 community mobilization project, which is funded by the Bill & Melinda Gates Foundation through Avahan, its India AIDS Initiative. The views expressed herein are those of the authors and do not necessarily reflect the official policy or position of the Bill & Melinda Gates Foundation and Avahan. The funders had no role in study design, data collection and analysis, decision to publish, or preparation of the manuscript.
